# Supplementary material for: Individualised mapping of living human brain mitochondria by MRI reveals signatures of bioenergetic defects
Source: bioRxiv. 2026 Jun 10:2026.06.09.730804. Preprint. [Version 1] doi: 10.64898/2026.06.09.730804 (PMC13277877; doi:10.64898/2026.06.09.730804)
Supplement: Supplementary file 1 [file NIHPP2026.06.09.730804v1-supplement-1.pdf]

bioRxiv preprint doi: https://doi.org/10.64898/2026.06.09.730804; this version posted June 10, 2026. The copyright holder for this preprint (which was not certified by peer review) is the author/funder, who has granted bioRxiv a license to display the preprint in perpetuity. It is made available under a CC-BY 4.0 International license.

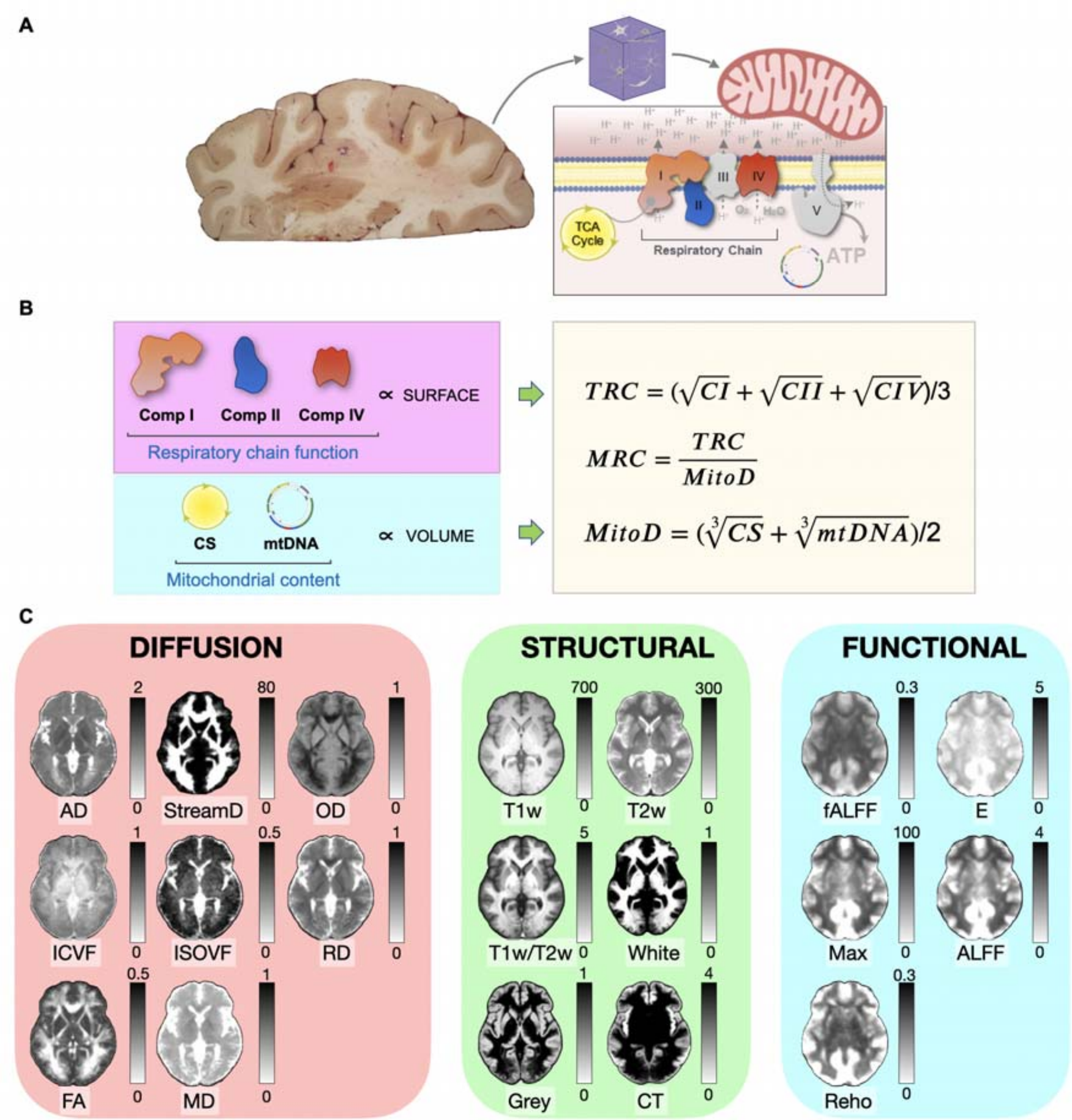

Supplementary Figure 1 | Biological and neuroimaging components underlying in vivo mapping of human brain mitochondrial features. (A) Overview of enzyme activity assays. *Left*, brain slab prior to voxelization.*Right*, schematic of the inner mitochondrial membrane illustrating respiratory chain complexes and ATP production. (B) Conceptual framework linking organelle geometry to mitochondrial function. Activities of respiratory complexes (CI, CII, and CIV) reflect mitochondrial surface-dependent processes, whereas citrate synthase (CS) activity and mtDNA copy number index mitochondrial volume. These measures are combined to derive tissue respiratory capacity (TRC) and mitochondrial density (MitoD). (C) Group-averaged diffusion, structural, and functional MRI templates derived from a subset of 10 healthy participants, approximately matched to the post-mortem brain sample in sex (all males) and age (36-54 years). Diffusion-weighted imaging metrics include axial diffusivity (AD), fractional anisotropy (FA), perpendicular diffusivity (RD), mean diffusivity (MD), streamline density (STreamD), intra-cellular volume fraction (ICVF), isotropic volume fraction (ISOVF), and orientation dispersion index (OD°. Structural MRI metrics include T1-weighted (T1w), T2-weighted (T2w), T1w/T2w ratio, cortical

bioRxiv preprint doi: https://doi.org/10.64898/2026.06.09.730804; this version posted June 10, 2026. The copyright holder for this preprint (which was not certified by peer review) is the author/funder, who has granted bioRxiv a license to display the preprint in perpetuity. It is made available under a CC-BY 4.0 International license.

thickness (CT), and probabilistic maps of grey matter (GM) and white matter (WM). Functional %RI metrics include amplitude of low-frequency fluctuations (ALFF), fractional ALFF (fALFF), regional homogeneity (ReHo), maximum BOLD amplitude (Max), and entropy-based measures of signal complexity (E). These multimodal templates served as predictors in the regression models used to estimate voxel-wise mitochondrial features.

bioRxiv preprint doi: https://doi.org/10.64898/2026.06.09.730804; this version posted June 10, 2026. The copyright holder for this preprint (which was not certified by peer review) is the author/funder, who has granted bioRxiv a license to display the preprint in perpetuity. It is made available under a CC-BY 4.0 International license.

	Abbreviation	MRI metric	CI	CII	CIV	MitoD	TRC	MRC
Diffusion MRI	AD	axial water diffusivity		.250	.281		.231	.213
	FA	fractional anisotropy (white matter density)				.502		
	RD	radial water diffusivity	-.196	-.378	-.442		-.419	-.420
	StreDensity	streamlines density				-.119		
	ICVF	intra-cellular volume fraction						-.102
	ISOCF	extra-cellular volume fraction						
	MD	mean water diffusivity						
	OD	orientation dispersion index (neurite complexity)	.146	.238	.243	.297	.252	.344
Structural MRI	T1W	T1w imaging	-.182			.229		-.219
	T2W	T2w imaging	-.214			-.252		
	T1w/T2w	T1w/T2w ratio		.209				
	CT	cortical thickness				-.130		
	GM	probability of gray matter	-.217					
	WM	probability of white matter	-.655	-.565	.459	-.907	-.442	-.154
Functional MRI	Max_activity	maximum bold derived from fMRI		-.100	-.192		-.168	
	Reho	regional homogeneity	-.264				-.187	-.266
	Entropy	synaptic complexity derived from fMRI	-.445					
	ALFF	amplitude of Low-Frequency Fluctuation	.454					
	fALFF	ratio between low and and high frequency fluctuations	.435	.316	.325	.273	.526	.421

Supplementary Table 1 | List of neuroimaging metrics and their standardized beta coefficient relationship with the mitochondrial features.

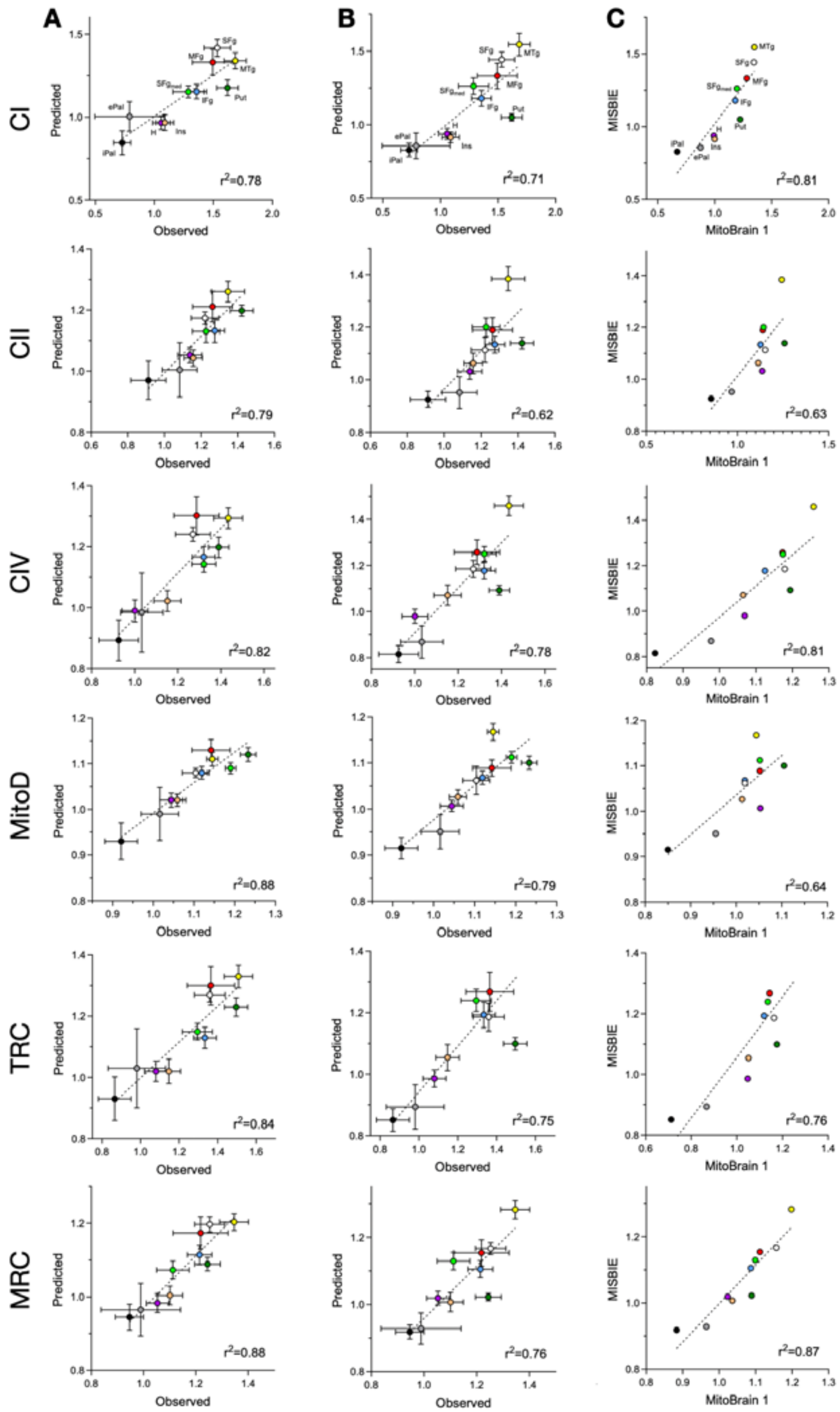

Supplementary Figure 2 | Cross-dataset correspondence of in vivo mitochondrial feature mapping. (A) Regional correlations between observed post-mortem mitochondrial measurements and MRI-predicted mitochondrial features, averaged across cortical and subcortical regions, as reported in Mosharov et al. 2025. Error bars indicate the standard error of the mean across regions. (B) Replication of the same correlations in the independent neuroimaging dataset acquired in the present study. (C) Direct correspondence between regional mitochondrial feature predictions from Mosharov et al. 2025 and those derived from the current dataset, demonstrating cross-study consistency in the predicted mitochondrial maps.

bioRxiv preprint doi: https://doi.org/10.64898/2026.06.09.730804; this version posted June 10, 2026. The copyright holder for this preprint (which was not certified by peer review) is the author/funder, who has granted bioRxiv a license to display the preprint in perpetuity. It is made available under a CC-BY 4.0 International license.

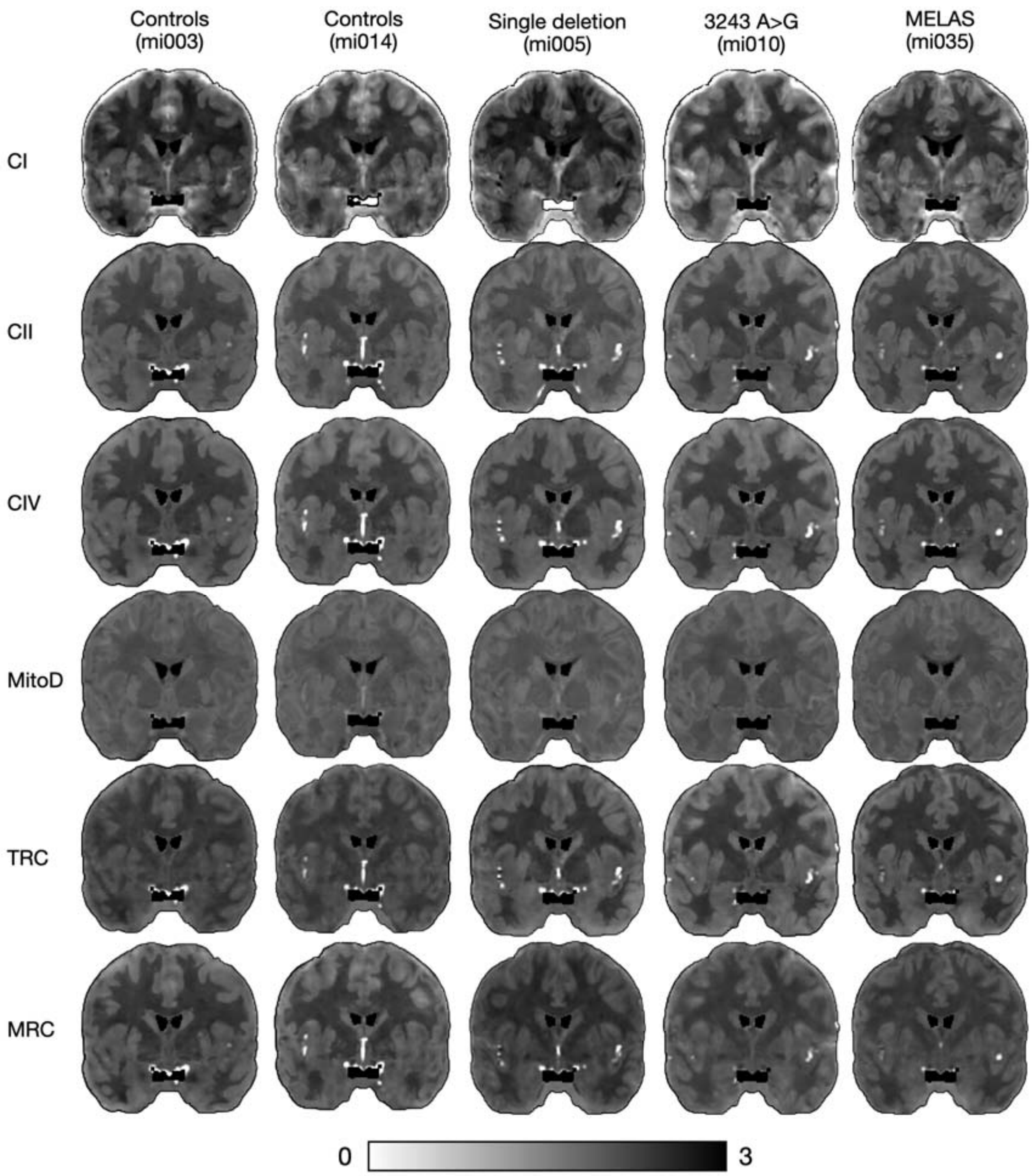

Supplementary Figure 3 | Examples of Controls and patients' predicted mitochondrial maps

bioRxiv preprint doi: https://doi.org/10.64898/2026.06.09.730804; this version posted June 10, 2026. The copyright holder for this preprint (which was not certified by peer review) is the author/funder, who has granted bioRxiv a license to display the preprint in perpetuity. It is made available under a CC-BY 4.0 International license.

	Mito Feature	F	p value
Grey Matter	CI	0.65	0.58
	CII	4.48	0.01
	CIV	1.81	0.15
	MitoD	3.58	0.02
	MRC	0.40	0.76
	TRC	2.85	0.04
White Matter	CI	0.81	0.49
	CII	4.82	0.00
	CIV	1.24	0.30
	MitoD	4.47	0.01
	MRC	1.61	0.19
	TRC	1.21	0.31
Mixed	CI	0.47	0.70
	CII	4.73	0.00
	CIV	1.09	0.36
	MitoD	4.51	0.01
	MRC	0.59	0.62
	TRC	1.85	0.15

Supplementary Table 2 | Group effects (F statistics from MANCOVA), corrected for age, for each mitochondrial feature across the grey, white, and mixed matter, excluding the MELAS group, with corresponding p values.

bioRxiv preprint doi: https://doi.org/10.64898/2026.06.09.730804; this version posted June 10, 2026. The copyright holder for this preprint (which was not certified by peer review) is the author/funder, who has granted bioRxiv a license to display the preprint in perpetuity. It is made available under a CC-BY 4.0 International license.

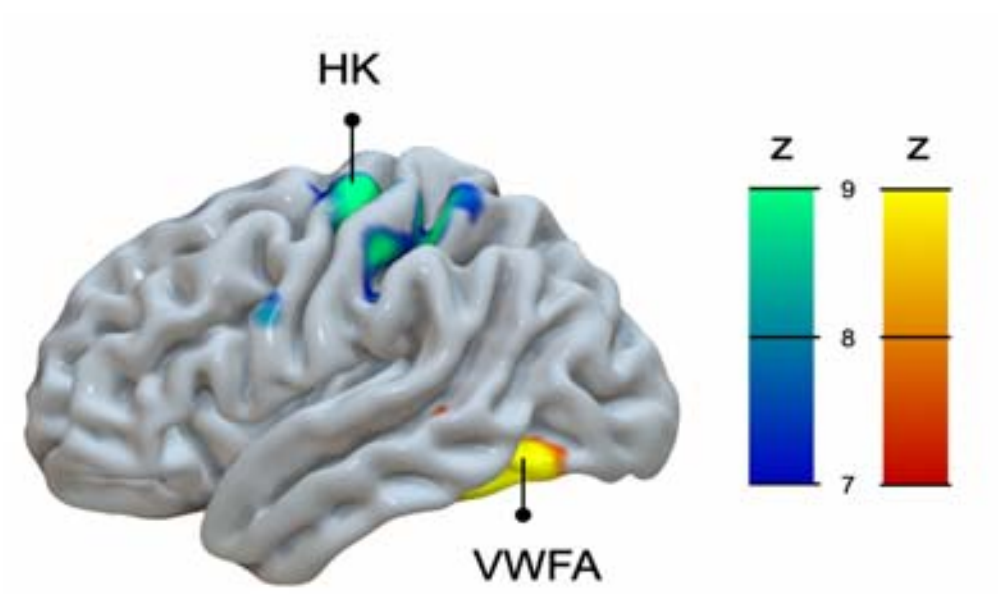

Supplementary Figure 4 | Typical functional activations of the hand-knob and visual word form area. Meta-analytic activation maps illustrate typical cortical responses associated with hand movement tasks, highlighting the hand knob region (in cyan, HK) and reading tasks, activating the visual word form area (in yellow, VWFA). Maps were derived from the Neurosynth meta-analytic database (https://neurosynth.org) and displayed as Z-score maps summarizing consistent task-related activations across studies[35].

bioRxiv preprint doi: https://doi.org/10.64898/2026.06.09.730804; this version posted June 10, 2026. The copyright holder for this preprint (which was not certified by peer review) is the author/funder, who has granted bioRxiv a license to display the preprint in perpetuity. It is made available under a CC-BY 4.0 International license.

	Mito Feature	Fasting	-5 min	5 min	10 min	20 min	30 min	60 min	90 min	120 min
Grey Matter	CI	0.14 (0.32)	-0.10 (0.29)	-0.12 (0.30)	-0.15 (0.33)	-0.22 (0.42)	-0.18 (0.36)	-0.20 (0.38)	-0.13 (0.31)	-0.15 (0.33)
	CII	0.22 (0.41)	0.28 (0.56)	0.28 (0.56)	0.28 (0.55)	0.24 (0.45)	0.29 (0.58)	0.23 (0.43)	0.24 (0.46)	0.26 (0.49)
	CIV	0.09 (0.29)	-0.10 (0.29)	-0.10 (0.29)	-0.08 (0.28)	-0.19 (0.37)	-0.11 (0.30)	-0.15 (0.33)	-0.12 (0.31)	-0.14 (0.32)
	MitoD	*0.24 (0.46)*	*0.46 (2.22)*	*0.46 (2.31)*	*0.47 (2.59)*	0.45 (1.69)	0.49 (2.79)	0.43 (1.53)	0.43 (1.53)	0.46 (2.14)
	MRC	-0.07 (0.28)	-0.48 (3.02)	-0.49 (3.49)	-0.54 (6.19)	-0.57 (7.02)	-0.54 (5.63)	*-0.57 (6.52)*	*-0.51 (3.53)*	*-0.57 (7.85)*
	TRC	0.13 (0.32)	-0.03 (0.27)	-0.02 (0.27)	-0.03 (0.27)	-0.10 (0.30)	-0.04 (0.27)	-0.09 (0.29)	-0.06 (0.28)	-0.08 (0.28)
White Matter	CI	0.22 (0.42)	-0.13 (0.31)	-0.15 (0.33)	-0.19 (0.37)	-0.26 (0.50)	-0.26 (0.50)	-0.24 (0.44)	-0.17 (0.35)	-0.18 (0.36)
	CII	0.44 (1.80)	*0.72 (236.08)*	*0.72 (207.6)*	*0.70 (128.29)*	*0.72 (104.94)*	*0.74 (248.35)*	*0.69 (58.50)*	*0.70 (98.09)*	*0.74 (283.14)*
	CIV	0.09 (0.29)	0.04 (0.27)	0.01 (0.27)	0.04 (0.27)	-0.18 (0.36)	-0.06 (0.28)	-0.09 (0.30)	-0.04 (0.27)	0.01 (0.27)
	MitoD	0.52 (3.91)	0.74 (322.73)	0.74 (328.1)	0.71 (144.64)	0.73 (154.29)	0.74 (294.84)	0.73 (141.77)	0.72 (153.30)	0.75 (370.39)
	MRC	-0.13 (0.31)	-0.54 (6.22)	-0.55 (7.43)	-0.59 (12.54)	-0.62 (15.41)	-0.62 (19.93)	-0.61 (11.81)	-0.57 (8.52)	-0.59 (11.73)
	TRC	0.27 (0.53)	0.10 (0.29)	0.10 (0.29)	0.09 (0.28)	-0.05 (0.28)	0.00 (0.27)	-0.02 (0.28)	0.02 (0.27)	0.04 (0.28)
Mixed	CI	0.25 (0.48)	-0.08 (0.28)	-0.10 (0.29)	-0.14 (0.32)	-0.21 (0.40)	-0.20 (0.39)	-0.18 (0.36)	-0.12 (0.31)	-0.14 (0.33)
	CII	0.49 (3.08)	0.66 (52.38)	0.66 (48.53)	0.68 (70.34)	0.67 (33.29)	0.67 (44.34)	0.63 (18.21)	0.64 (24.32)	0.66 (41.19)
	CIV	0.18 (0.36)	-0.03 (0.27)	-0.04 (0.27)	-0.01 (0.26)	-0.17 (0.35)	-0.08 (0.29)	-0.09 (0.29)	-0.05 (0.28)	-0.08 (0.29)
	MitoD	0.47 (2.37)	0.68 (84.09)	0.69 (86.79)	0.69 (100.21)	0.68 (47.08)	0.70 (89.95)	0.67 (36.91)	0.66 (39.57)	0.69 (68.46)
	MRC	0.01 (0.27)	-0.48 (3.10)	-0.50 (3.62)	-0.54 (5.84)	-0.56 (6.43)	-0.57 (8.02)	-0.54 (4.88)	-0.51 (3.58)	-0.56 (7.04)
	TRC	0.32 (0.70)	0.04 (0.27)	0.04 (0.27)	0.05 (0.27)	-0.06 (0.28)	-0.02 (0.27)	-0.02 (0.28)	0.00 (0.27)	-0.03 (0.27)

Supplementary Table 3 | Predicted mitochondrial features and their relationship with plasma GDF15 at multiple time points surrounding the stress task. Pearson r values are shown in each cell, with corresponding Bayes factor (BF_{10}) in brackets.

bioRxiv preprint doi: https://doi.org/10.64898/2026.06.09.730804; this version posted June 10, 2026. The copyright holder for this preprint (which was not certified by peer review) is the author/funder, who has granted bioRxiv a license to display the preprint in perpetuity. It is made available under a CC-BY 4.0 International license.

Neuropsychological Scores/PCs	PC1	PC2	PC3	PC4	PC5	PC6	PC7	PC8	PC9	PC10
Screening Numbers & Letters Part A Efficiency	**0.64**	0.02	0.01	0.01	0.00	0.00	0.04	0.07	0.02	0.01
Screening Numbers & Letters Part A Speed	**0.63**	0.00	0.00	0.01	0.00	0.00	0.09	0.11	0.02	0.02
Condition1: Color Naming	**0.57**	0.08	0.01	0.04	0.05	0.01	0.02	0.01	0.00	0.00
Condition2: Word Reading	**0.56**	0.10	0.00	0.07	0.05	0.00	0.01	0.00	0.01	0.00
RBANS Total Score	**0.49**	0.07	0.00	0.01	0.04	0.04	0.00	0.05	0.00	0.00
Condition3: Inhibition	**0.45**	0.02	0.12	0.04	0.08	0.01	0.00	0.02	0.00	0.00
Condition3: Letter Sequencing	**0.40**	0.08	0.01	0.01	0.20	0.01	0.01	0.00	0.04	0.11
Condition4: Number-Letter Switching	**0.29**	0.08	0.19	0.03	0.16	0.02	0.02	0.00	0.01	0.02
Condition 1: Category Switching Total Switching Accuracy	0.00	**0.73**	0.04	0.00	0.00	0.00	0.03	0.01	0.02	0.00
Condition 1: Category Switching Total Correct	0.01	**0.69**	0.07	0.00	0.00	0.00	0.04	0.01	0.04	0.00
Condition 1: Category Fluency Total Correct	0.08	**0.62**	0.00	0.02	0.04	0.01	0.01	0.00	0.01	0.00
Condition 1: Letter Fluency Total Correct	0.11	**0.39**	0.01	0.03	0.03	0.08	0.02	0.00	0.00	0.01
RBANS List Learning Total Score	0.02	0.15	**0.02**	0.03	0.14	0.10	0.01	0.08	0.00	0.08
Matrix Reasoning Raw Score	0.01	0.00	**0.71**	0.00	0.00	0.06	0.02	0.00	0.01	0.00
Confirmed Correct Sorts	0.02	0.06	**0.56**	0.00	0.06	0.02	0.02	0.02	0.01	0.00
RBANS Line Orientation Total Score	0.00	0.00	**0.47**	0.00	0.01	0.04	0.00	0.03	0.02	0.04
Vocabulary raw score	0.01	0.14	**0.34**	0.18	0.03	0.00	0.00	0.03	0.02	0.05
Screening Digital Forward Longest Span	0.02	0.00	0.00	**0.84**	0.01	0.03	0.00	0.00	0.01	0.00
Screening Digital Forward	0.02	0.00	0.00	**0.82**	0.01	0.06	0.00	0.00	0.00	0.00
TOPF Total	0.07	0.17	0.21	**0.24**	0.00	0.01	0.01	0.00	0.06	0.00
RBANS Picture Naming Total Score	0.07	0.06	0.10	**0.23**	0.01	0.01	0.02	0.01	0.02	0.00
Screening Numbers & Letters Part A Error	0.01	0.00	0.00	0.00	**0.70**	0.00	0.02	0.00	0.02	0.00
Condition2: Number Sequencing	0.14	0.07	0.01	0.03	**0.32**	0.00	0.02	0.05	0.05	0.02
Screening Digital Backward Longest Span	0.02	0.01	0.06	0.03	0.01	**0.81**	0.00	0.01	0.00	0.00
Screening Digital Backward	0.02	0.00	0.07	0.04	0.00	**0.80**	0.00	0.00	0.00	0.00
Screening Numbers & Letters Part B Efficiency	0.04	0.02	0.02	0.00	0.01	0.00	**0.81**	0.00	0.00	0.01
NAB screening numbers and letters part b speed	0.10	0.04	0.01	0.00	0.05	0.00	**0.68**	0.01	0.01	0.00
Screening Shape Learning Immediate Recognition	0.04	0.01	0.03	0.00	0.00	0.00	0.01	**0.72**	0.10	0.00
Screening Shape Learning Delayed Recognition	0.04	0.01	0.04	0.00	0.00	0.01	0.00	**0.51**	0.15	0.03
Screening Shape Learning Percent Retetion	0.00	0.01	0.01	0.00	0.02	0.01	0.02	0.01	**0.77**	0.02
Condition4: Inhibition/Switching	0.07	0.02	0.03	0.09	0.11	0.01	0.02	0.05	**0.22**	0.11
NAB screening numbers and letters part b errors	0.00	0.00	0.00	0.01	0.01	0.00	0.01	0.02	0.01	**0.67**

Supplementary Table 4 | List of neuropsychological scores and their squared standardized principal component analysis loadings. Rows are colored/bold according to their dominant principal component.
